# *QuickStats:* Age-Adjusted Alcohol-Induced Death Rates* Among Adults Aged ≥45 Years, by Sex — United States, 2014–2024

**DOI:** 10.15585/mmwr.mm7532a3

**Published:** 2026-08-20

**Figure:**
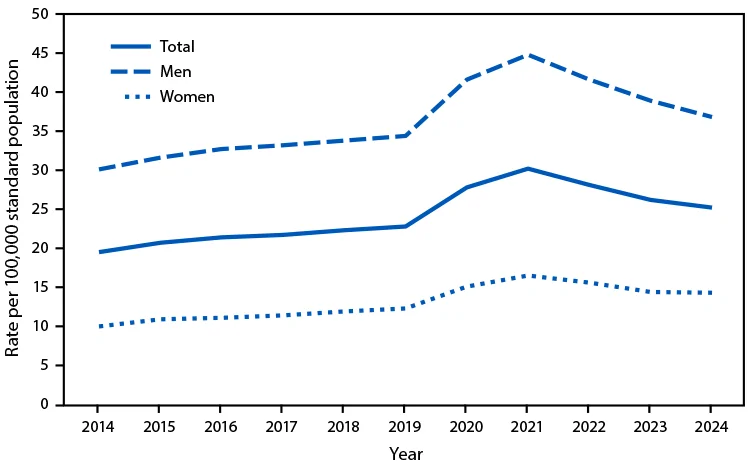
From 2014 to 2024, the age-adjusted death rate for alcohol-induced deaths among adults aged ≥45 years increased from 19.5 to 25.2. Death rates increased among men and women and were higher for men (36.8) than women (14.3) in 2024 and throughout the period.

**Supplementary Table:** https://stacks.cdc.gov/view/cdc/258536#tabs-3

**Source:** National Center for Health Statistics, National Vital Statistics System, Mortality Data

